# Retraction Note: Myocardial ischemia reperfusion injury is alleviated by curcumin-peptide hydrogel via upregulating autophagy and protecting mitochondrial function

**DOI:** 10.1186/s13287-022-02732-3

**Published:** 2022-02-03

**Authors:** Chi-Lin Liao, Yang Liu, Meng-Zhao Huang, Hua-Yong Liu, Zi-Liang Ye, Qiang Su

**Affiliations:** 1 Department of Cardiology, People’s Hospital of Baise, Baise, 533000 People’s Republic of China; 2 Department of Cardiology, The Second People’s Hospital of Nanning City, The Third Affiliated Hospital of Guangxi Medical University, Nanning, 530031 People’s Republic of China; 3 Department of Cardiology, Affiliated Hospital of Guilin Medical University, No. 15, Lequn Road, Xiufeng District, Guilin, 541001 Guangxi Zhuang Autonomous Region People’s Republic of China

## Retraction Note: Stem Cell Research & Therapy (2021) 12:89 10.1186/s13287-020-02101-y

The authors have retracted this article. After publication the authors re-examined their data and found that the statistics for LC3-II/I in Figure 4B and p-Cx43 in Figure 5B were incorrect due to an input error during the calculations.

All authors agree to this retraction.

